# Metabolomics of *Eothenomys miletus* from five Hengduan Mountains locations in summer

**DOI:** 10.1038/s41598-019-51493-2

**Published:** 2019-10-17

**Authors:** Hai-ji Zhang, Zheng-kun Wang, Wan-long Zhu

**Affiliations:** 1 Key Laboratory of Adaptive Evolution and Ecological Conservation on Plants and Animals in Southwest Mountain Ecosystem of Yunnan Higher Education Institutes, School of Life Sciences, Yunnan Normal University, Kunming, 650500 People’s Republic of China; 2 Yunnan Normal University, Engineering Research Center of Sustinable Development and Utilization of Biomass Energy Ministry of Education, Kunming, 650500 People’s Republic of China; 3 Key Laboratory of Yunnan Province for Biomass Energy and Environment Biotechnology, Kunming, 650500 People’s Republic of China

**Keywords:** Metabolomics, Environmental chemistry

## Abstract

Climatic characteristics of Hengduan Mountains region were diverse, and *Eothenomys miletus* was a native species throughout this region. To investigate adaptive strategies of *E*. *miletus* to environmental factors in different locations in this region, five locations were selected, including Deqin (DQ), Xianggelila (XGLL), Lijiang (LJ), Jianchuan (JC) and Ailaoshan (ALS). Then, body mass, visceral organ masses, and serum and liver metabolomes of *E*. *miletus* from each location were examined. The results showed that body mass was significantly different among these five sites. Liver mass was lower in ALS than in other locations. PLS-DA analysis, metabolite tree maps and heat maps of serum and liver metabolites showed that samples from DQ and XGLL clustered together, as did the samples from LJ, JC and ALS. Serum concentrations of lipid and amino acid metabolites, concentrations of TCA cycle intermediates, lipid metabolites and amino acid metabolites in livers from DQ and XGLL were higher than those from other three regions. However, the concentrations of glycolytic metabolites were lower in DQ and XGLL. All these results indicated that *E*. *miletus* adapts to changes in environmental temperature and altitude of this region by adjusting body mass and serum and liver metabolite concentrations.

## Introduction

Metabolomics aims to detect, identify and quantify all metabolites in biological samples^1,2^. Metabolomics can quantitatively measure multiparameter metabolic responses in living systems by monitoring hundreds of low-molecular-weight metabolites simultaneously^3^ and thus assess differences in metabolite levels^4^. The most commonly used samples in metabolomic analyses were serum, cell and tissue extracts^5^. Many studies of serum and liver metabolomics were available^6,7^; for example, the environmental metabolomics of Mongolian gerbils (*Meriones unguiculatus*) was revealed by measuring serum metabolites^8^, and the concentration of liver metabolites in Marwari goats can reflect extreme environmental conditions^9^. Moreover, studies have shown that variations in liver glucose reflect changes in energy metabolism^10^. Environmental metabolomics is the application of metabolomics to the study of interactions between organisms and the environment^11^. Cold adaptation causes changes in the metabolism of *Drosophila melanogaster*, altering the levels of some sugars, polyamines and metabolic intermediates^12^; warmer temperatures lead to reduced concentrations of several amino acids (glutamine, tyrosine and phenylalanine) and changes in lipid metabolism in Atlantic salmon^13^. Therefore, changes in environmental temperature and other factors are thought to cause specific metabolic changes in mammals.

Physiological regulations of body mass and energy metabolism are the main strategies used by small mammals to cope with environmental changes^14^. In addition, phenotypic plasticity in body composition and the digestive system are adaptive characteristics of animals^15^ that have an important influence on nutrition acquisition and energy utilization efficiency^16,17^. For example, seasonal variations occur in body mass of *Cettia cetti*^18^, while *Lasiopodomys brandtii* has a heavier liver and gastrointestinal tract under cold acclimation^19^.

The Yunnan red-backed vole, *Eothenomys miletus* (Mammalia: Rodentia: Microtus), is a native species of the Hengduan Mountains region^20^. *E*. *miletus* is reported to show seasonal variations in body mass and to increase its metabolic rate and reduce its serum leptin levels under cold exposure or short photoperiod conditions^21–23^. However, changes in body mass and metabolome of *E*. *miletus* in different locations in the Hengduan Mountains region have not been reported. In the present study, we examined body mass, visceral organ masses, and serum and liver metabolomes in this species. We hypothesize that *E*. *miletus* in different locations can adjust these traits to adapt to the differences in environmental temperature and altitude across the Hengduan Mountains region. We predicted that *E*. *miletus* would show lower body mass and higher lipid and amino acid metabolite concentrations in cold locations.

## Materials and Methods

### Ethics statement

This research was performed in accordance with the NIH *Guide for the Principles of Animal Care*. The protocol and study were approved by the Animal Care and Use Committee of the School of Life Sciences, Yunnan Normal University (No. 13-0901-011). All researchers and students were certified before performing animal studies. Permits were obtained from all local authorities to capture the animals from the five locations.

### Animals and experimental designs

*E*. *miletus* were captured from five locations in the Hengduan Mountains region in the summer of 2016 (Table 1). From north to south, the animals were collected from Deqin (DQ), Xianggelila (XGLL), Lijiang (LJ), Jianchuan (JC) and Ailaoshan (ALS). All the experimental animals were healthy adults in the nonreproductive stage. The sample size and climate information for each location are detailed in Table 1.

**Table 1:** Coordinates, altitudes, annual mean temperatures, summer temperatures, precipitation and vegetation types of five regions containing *Eothenomys miletus*.

Region	Sample number	Site	Altitude	Annual average temperature	Summer temperature	Precipitation	Vegetation types
DQ	7 (4♂ 3♀)	99°03′75″E, 28°35′14″N	3459 m	4.7 °C	17.2 °C	633.7 mm	Alpine meadow
XGLL	12 (6♂ 6♀)	99°83′16″E, 27°90′73″N	3321 m	5.5 °C	18.7 °C	984.2 mm	Subalpine meadow
LJ	12 (7♂ 5♀)	100°22′90″E, 26°87′53″N	2478 m	12.6 °C	21.0 °C	975.0 mm	Subalpine meadow and shrub
JC	12 (6♂ 6♀)	99°75′03″E, 26°43′95″N	2590 m	13.9 °C	23.7 °C	987.3 mm	Lobular shrub
ALS	12 (6♂ 6♀)	100°42′49″E, 24°90′30″N	2217 m	19.7 °C	27.8 °C	597.0 mm	Savanna shrub and grass

The animals were brought back to the local epidemic prevention station to remove fleas, determine body mass (accuracy of 0.01 g), and record sex and reproductive status. All animals were killed by decapitation. The blood was centrifuged at 4,000 rpm for 30 min after a 30 min interval. The liver was dissected immediately and placed in a cryopreservation tube. The serum and liver were stored in liquid nitrogen.

### Morphology

After collection of the trunk blood, the visceral organs, including the liver, heart, lung, kidneys, and spleen were extracted and weighed (±1 mg). All the organs (excluding the liver) were dried to a constant mass in an oven at 60 °C (at least 72 h) and then weighed again to obtain the dry mass.

### GC–MS detection

Liver samples (100 mg) were placed into 2 ml centrifuge tubes, and 1,000 μl of 80% methanol (pre-cooled at −20 °C) and five steel balls were added. The tubes were homogenized in a high flux bead mill at 70 Hz for 1 min. Then, 60 μl of 2-chloro-L-phenylalanine (0.2 mg/ml stock in methanol) and 60 μL of heptadecanoic acid (0.2 mg/ml stock in methanol) as an internal quantitative standard were added, and the tubes were vortexed for 60 s. Next, the tubes were placed into an ultrasound machine at room temperature for 30 min and then incubated for 30 min on ice. The samples were centrifuged for 10 min at 14,000 rpm and 4 °C, and 0.8 ml of supernatant was transferred into a new centrifuge tube and dried by vacuum concentration. Then, 60 μl of 15 mg/ml methoxyamine pyridine solution was added, and the tube was vortexed for 30 s and allowed to react for 120 min at 37 °C; 60 μl BSTFA reagent (containing 1% TMCS) was added to the mixture and incubated for 90 min at 37 °C. Finally, the samples were centrifuged at 12,000 rpm and 4 °C for 10 min, and the supernatant was transferred to a cuvette.

Briefly, 50 μl blood sample was added to an Eppendorf tube(1.5 ml), and 40 μl of methanol was added, fully blended for 1 min. Then, 60 μl of 2-chloro-L-phenylalanine (0.2 mg/ml stock in methanol) and 60 μL of heptadecanoic acid (0.2 mg/ml stock in methanol) as an internal quantitative standard were added, and the tubes were vortexed for 60 s. The supernatant was centrifuged for 10 minutes at 12 000 r/min at 4 °C. The supernatant was transferred to a new centrifugal tube of 1.5 ml. The sample was concentrated by a vacuum centrifugal concentrator. Adding 60 ul methoxyl solution to the reaction system, the reaction time was 2 h at 37 °C and the eddy oscillation time was 30 s. BSTFA reagent containing 1% trimethylchlorosilane was added to 60 ul. The reaction time was 90 min, 4 °C, 12 000 r/min and centrifuged for 10 min at 37 °C. The supernatant was added to the detection bottle to perform GC–MS analysis^24^.

The processed samples were detected and analysed based on Agilent GC–MS (7890A-5975C, CA, USA). The specific parameters of chromatographic conditions were as follows: HP-5MS capillary column(5% phenyl methyl silox: 30 m × 250 um i.d., 0.25-um; agilent J&W scientific, Folsom, CA); injection volume 1 ul, split injection, split ratio 20:1. The temperature of ion source is 250 °C, the temperature of inlet is 280 °C, and the temperature of interface is 150 °C. The initial temperature of the program was 70 °C, kept for 2 min, and rose to 300 °C at 10 °C/min for 5 min. The carrier gas is helium, the flow rate is 1 ml/min, and the total running time is 30 minutes. MS condition: electron bombardment ion (EI) source, electron energy 70 eV, full scanning mode; quadrupole scanning range m/z 35–780^25^.

### Statistical analysis

GC–MS data were analyzed by automated mass spectral deconvolution and identification system (AMDIS) software (NIST, CA, USA), which is compiled by the National Institute of Standards and Technology (NIST) mass spectral library. The original data is preprocessed by software XCMS (www.bioconductor.org/). Converting the original GC-MS data into CDF format. The XCMS program was used for peak identification, peak filtering and peak alignment to determine the parameters of XCMS. The three-dimensional matrices of retention time, plasmon-nucleus ratio and peak strength obtained above were combined with AMDIS program to annotate metabolites. The annotation databases were Wiley Registry Metabolites Database and NIST Commercial Database, in which the alkane retention index of metabolites is based on The Golm Metabolome Database(GMD) (http://gmd.mpimp-golm.mpg.de/), and the substance was further confirmed by the standard substance. The peak areas of each metabolite were normalized by the internal standard (2-chloro-L-phenylalanine and heptadecanoic acid). Quality control (QC) analysis can verify whether the system error of the whole experiment is within the controllable range. Then the processed data were imported into SIMCA-P software (Umetrics, Umea, Sweden), and multivariate statistical analysis was carried out, including PLS-DA analysis^24^. A metabolite tree map was constructed based on the Euclidean distance between samples, and clustering of samples was performed by a clustering algorithm. Hierarchical cluster analysis (HCA) was used to analyse the metabolite thermograms with the pheatmap function in the R package. Metabolic pathways were assigned to metabolites based on KEGG (http://www.genome.jp/kegg/)^26^, and Pathway Activity Profiling (PAPi) was used to compare the relative activities of different metabolic pathways in different groups (populations). All analysis was performed using the R package^27^. Differential metabolites were screened by one-way ANOVA analysis (P < 0.05) and ploidy change Log2 value (fold change >1.5 or fold change <0.667). The body mass and visceral organ mass data were analysed using the SPSS 15.0 software package. Prior to all statistical analyses, the data were examined for assumptions of normality and homogeneity of variance using the Kolmogorov–Smirnov and Levene tests, respectively. Body mass and visceral organ masses were analysed by one-way analysis of variance (ANOVA) or one-way analysis of covariance (ANCOVA), with body mass or carcass mass as a covariate, followed by the Tukey post hoc test. To detect possible associations of body mass with visceral organ masses, differential metabolites with environmental temperatures and altitudes, we used Pearson-correlation analysis. The results were presented as the means ± SEM, and P < 0.05 was considered to be statistically significant.

## Results

### Body mass and visceral organ mass

Differences in body mass were found among the five locations (*F = *3.54, *P < *0.05); body mass was higher in ALS and lower in DQ (Table 2). A remarkable difference in liver wet mass also existed among the five locations (*F = *3.895, *P < *0.01, Table 2), also with higher values in DQ and lower in ALS. No difference was found in the wet mass or dry mass of the other visceral organs (*P* > 0.05). Body mass was negatively correlated with liver wet mass (r = −0.61, P < 0.01), masses of other visceral organs were not related to body mass (P > 0.05).

**Table 2:** Body mass and visceral organ masses of *Eothenomys miletus* in five regions.

	DQ	XGLL	LJ	JC	ALS
Body mass (g)	32.63 ± 3.26^c^	33.73 ± 3.27^c^	36.11 ± 2.36^b^	37.69 ± 3.20^b^	40.25 ± 3.36^a^
Wet mass of heart (g)	0.19 ± 0.05^a^	0.19 ± 0.03^a^	0.22 ± 0.05^a^	0.21 ± 0.04^a^	0.19 ± 0.05^a^
Wet mass of liver (g)	1.96 ± 0.23^a^	1.76 ± 0.21^b^	1.65 ± 0.22^b^	1.71 ± 0.26^b^	1.36 ± 0.24^c^
Wet mass of spleen (g)	0.11 ± 0.06^a^	0.11 ± 0.04^a^	0.14 ± 0.07^a^	0.12 ± 0.08^a^	0.10 ± 0.02^a^
Wet mass of lung (g)	0.28 ± 0.05^a^	0.29 ± 0.04^a^	0.32 ± 0.05^a^	0.31 ± 0.03^a^	0.29 ± 0.06^a^
Wet mass of kidney (g)	0.41 ± 0.03^a^	0.45 ± 0.05^a^	0.48 ± 0.06^a^	0.42 ± 0.06^a^	0.44 ± 0.07^a^
Dry mass of heart (g)	0.05 ± 0.01^a^	0.05 ± 0.01^a^	0.06 ± 0.01^a^	0.05 ± 0.01^a^	0.05 ± 0.02^a^
Dry mass of spleen (g)	0.03 ± 0.01^a^	0.03 ± 0.01^a^	0.04 ± 0.02^a^	0.03 ± 0.02^a^	0.03 ± 0.01^a^
Dry mass of lung (g)	0.07 ± 0.01^a^	0.07 ± 0.02^a^	0.09 ± 0.03^a^	0.08 ± 0.01^a^	0.07 ± 0.01^a^
Dry mass of kidney (g)	0.11 ± 0.03^a^	0.13 ± 0.02^a^	0.14 ± 0.03^a^	0.12 ± 0.02^a^	0.11 ± 0.05^a^

Means with different superscript letters are significantly different (*P* < 0.05).

### Serum and liver metabolomics

**Figure 1:**
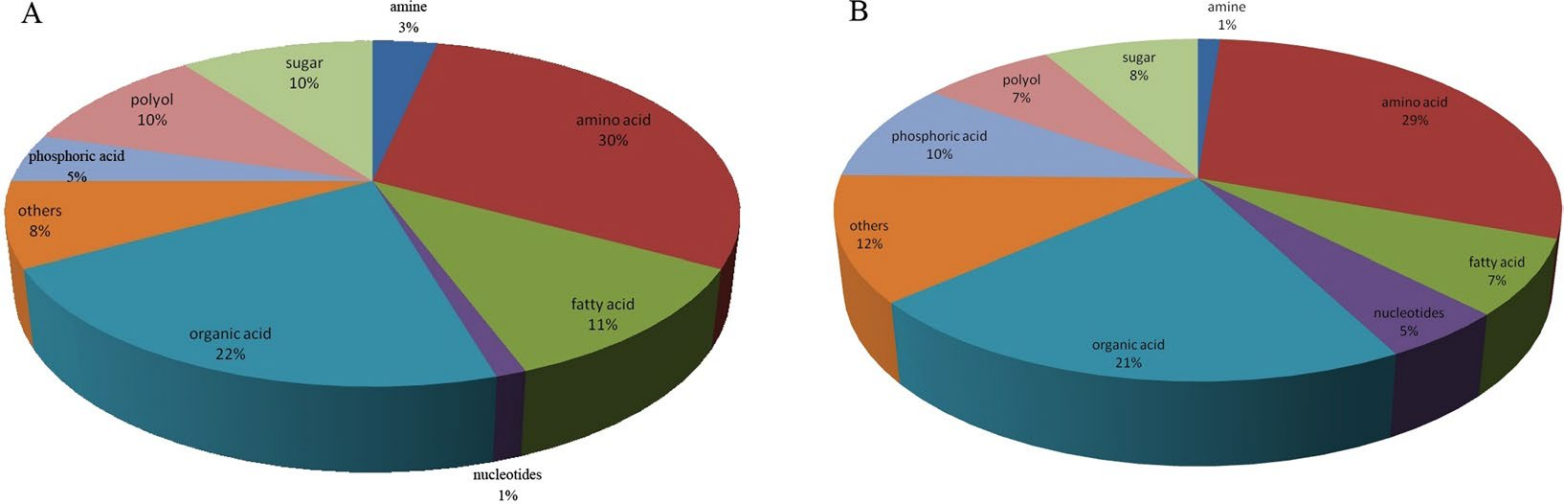
Classification of the serum (**A**) and liver (**B**) metabolites of *Eothenomys miletus*.

**Figure 2:**
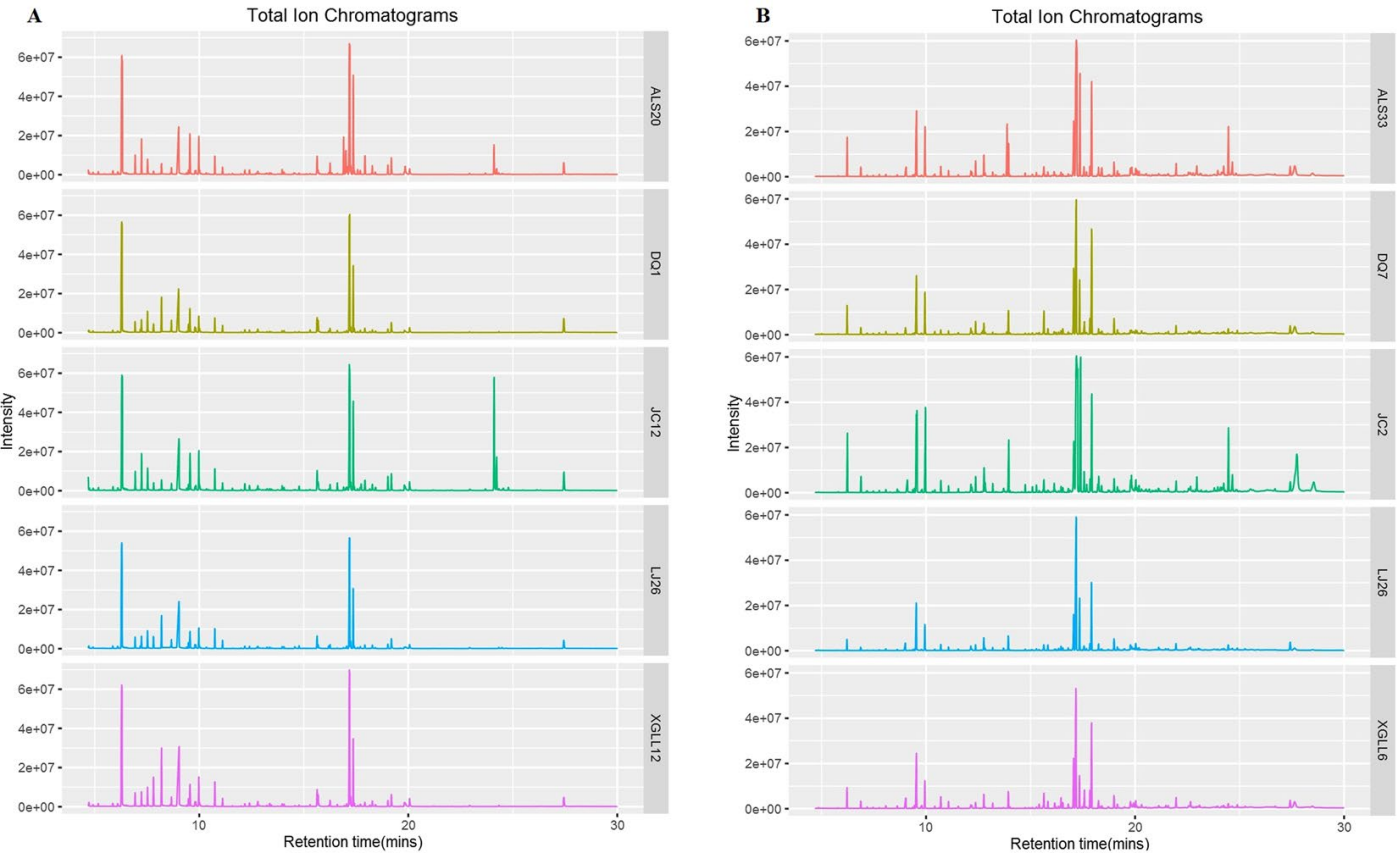
*Eothenomys miletus* serum (**A**) and liver (**B**) total ion current chromatograms.

A total of 88 metabolites were detected in the serum, and 86 metabolites were detected in the liver. The classification of the serum and liver metabolites in *E*. *miletus* is shown in Fig. 1. The total ionic current chromatograms of the serum and liver in the *E*. *miletus* groups are shown in Fig. 2 and reflect the differences among the study locations.

**Figure 3:**
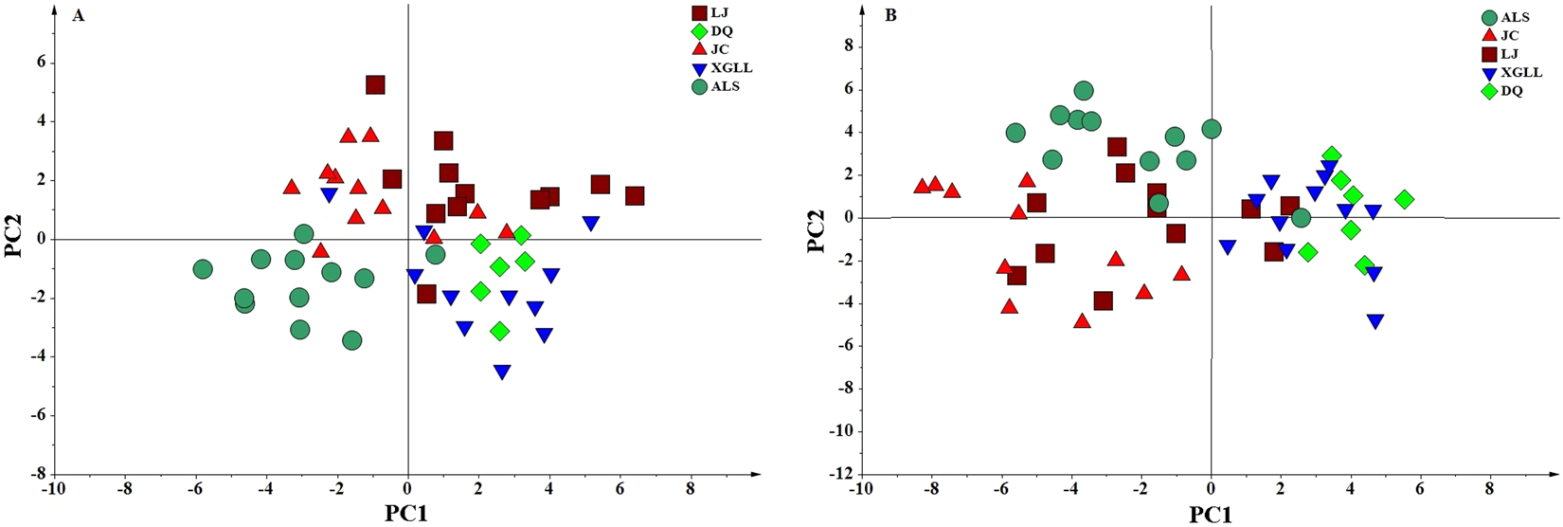
PLS-DA score plots of metabolites in *Eothenomys miletus* serum (**A**) and liver (**B**).

**Figure 4:**
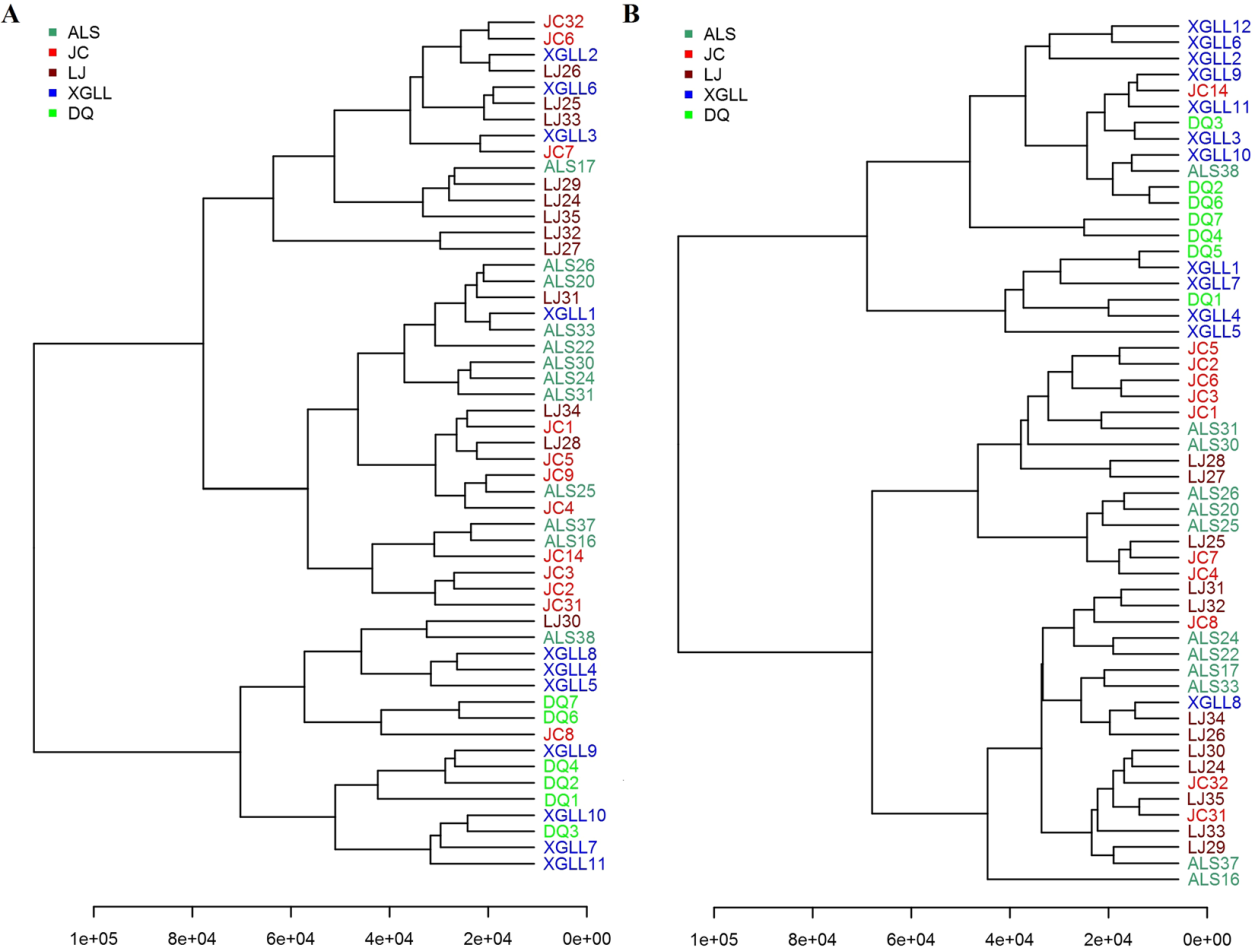
Serum metabolite tree map (**A**) and liver metabolite tree map (**B**) of *Eothenomys miletus*.

The results of the serum PLS-DA analysis showed that the XGLL and DQ populations clustered together, as did the JC, LJ and ALS populations (Fig. 3A); the liver metabolite PLS-DA analysis showed similar results (Fig. 3B). The tree map of serum metabolites showed that the DQ population and most of the XGLL population clustered together, while the LJ, JC and ALS populations were intermingled (Fig. 4A). The tree map of liver metabolites showed that the DQ population and XGLL population clustered together, and the LJ, JC and ALS populations clustered together (Fig. 4B).

**Figure 5:**
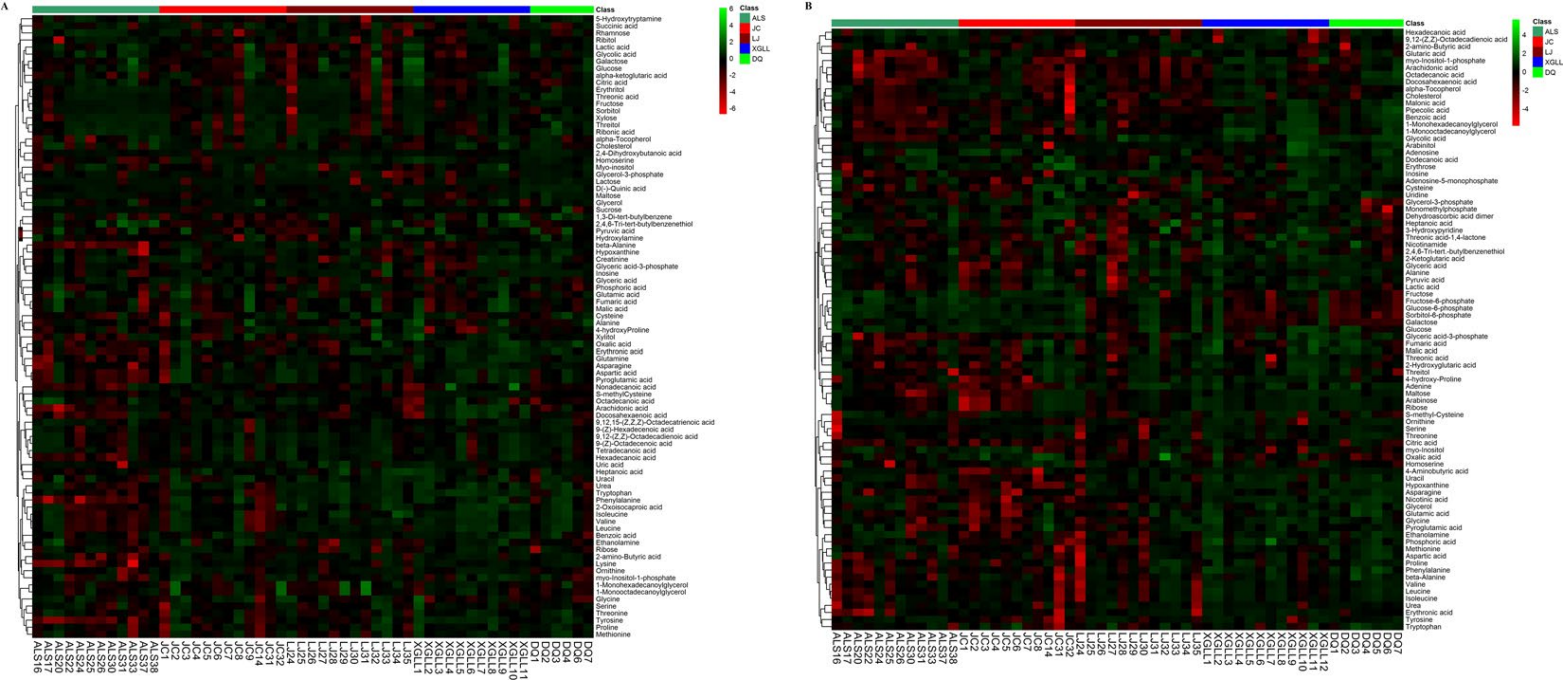
Heat map of serum metabolite differences (**A**) and liver metabolite differences (**B**) in *Eothenomys miletus*. Metabolites and sample two-way clustering heat maps. Colour depth represents metabolite contents in 5 populations of *Eothenomys miletus*. Green represents high content, and red represents low content.

**Figure 6:**
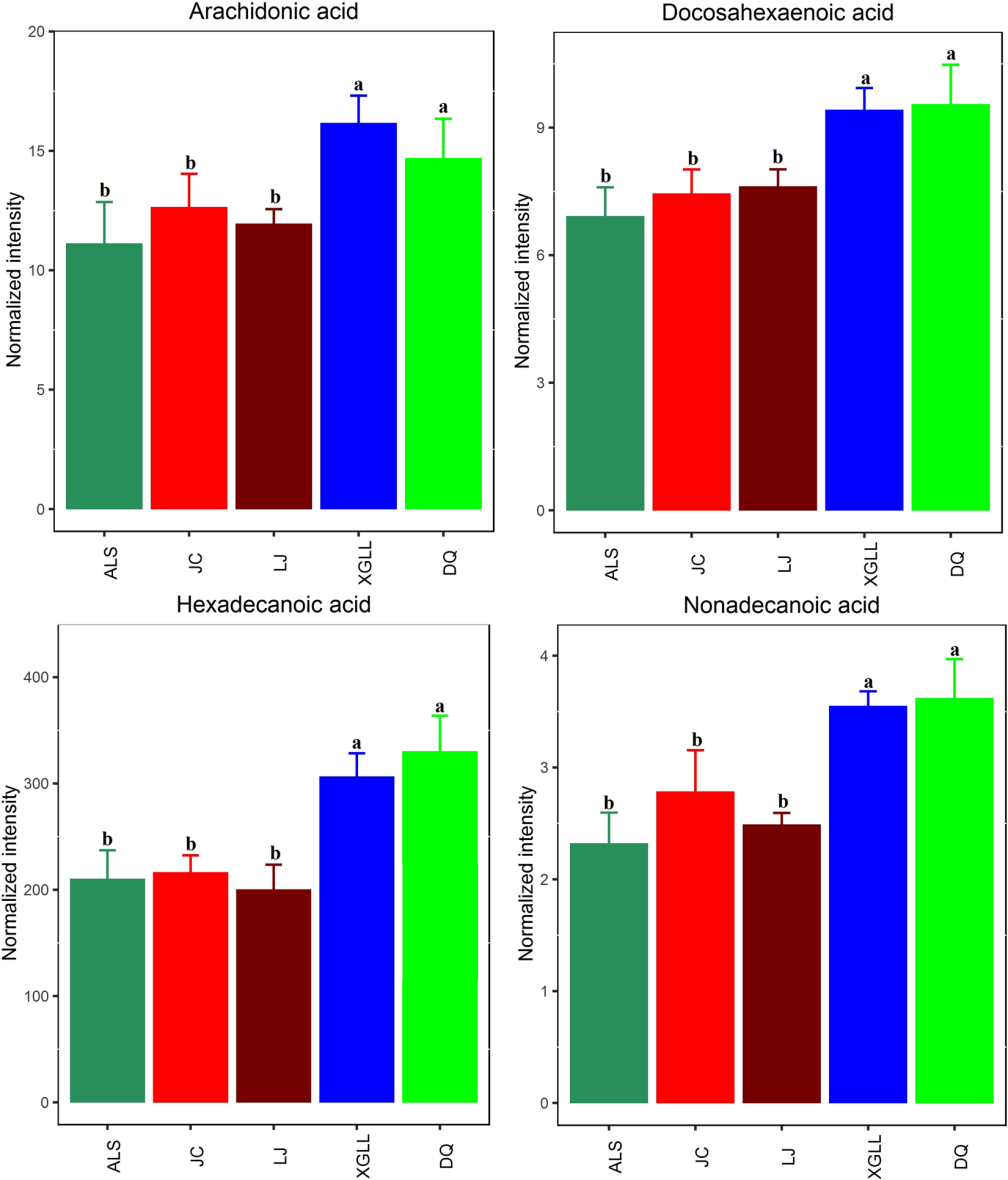
Lipid metabolite levels in *Eothenomys miletus* serum samples. Means with different superscript letters are significantly different (P < 0.05).

**Figure 7:**
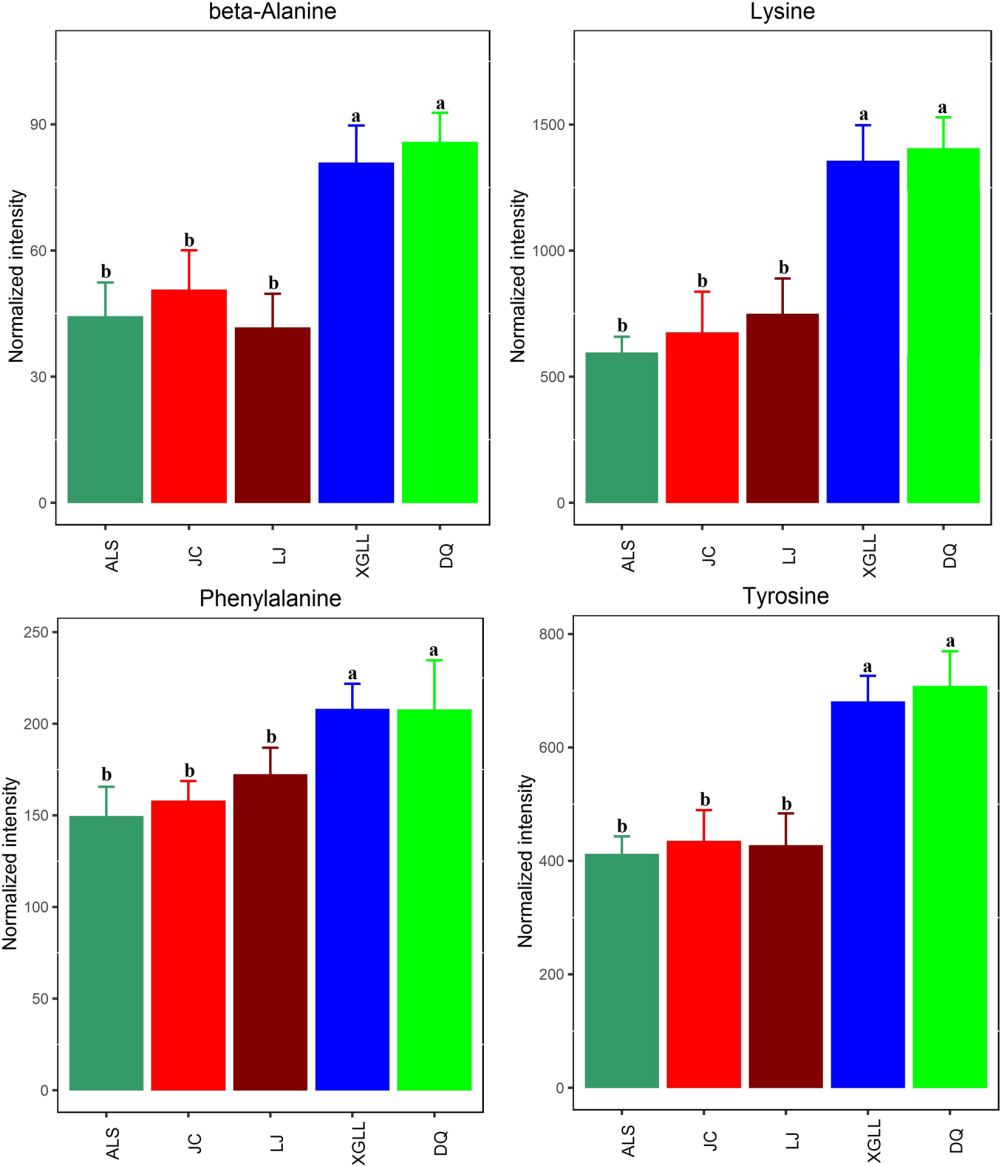
Amino acid metabolite levels in *Eothenomys miletus* serum samples. Means with different superscript letters are significantly different (P < 0.05).

**Figure 8:**
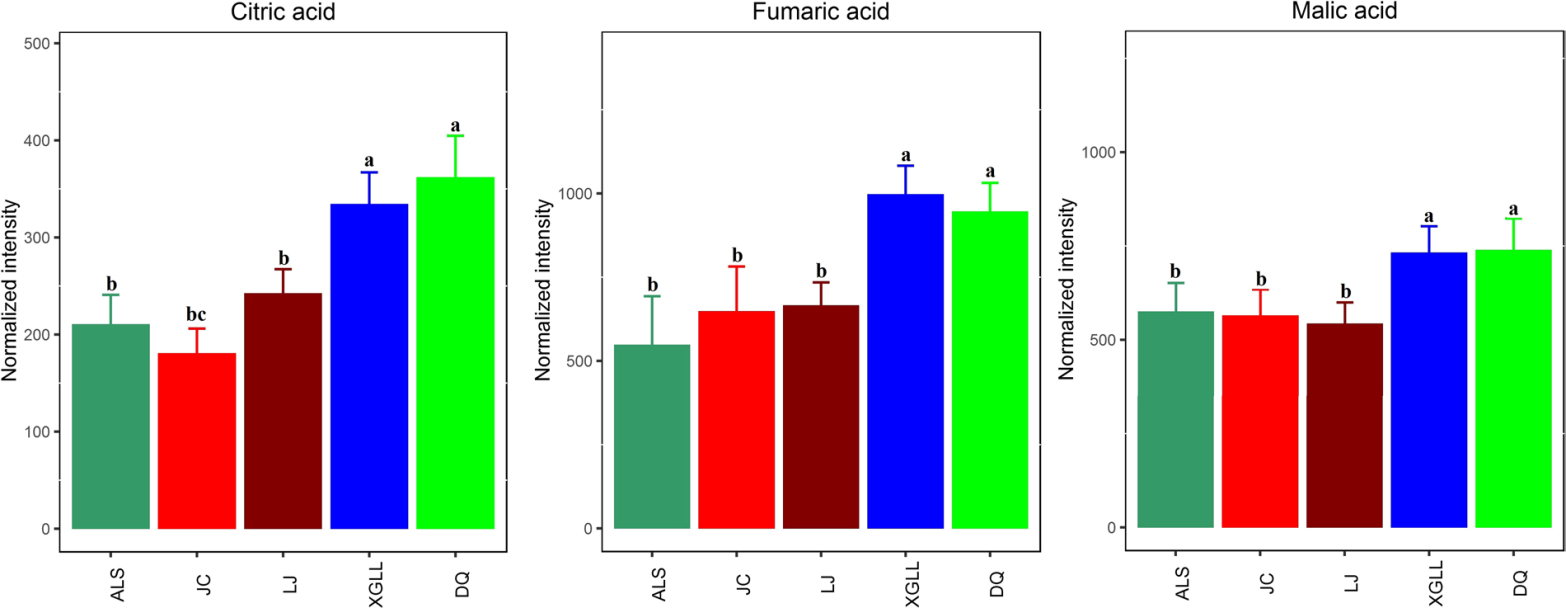
TCA cycle intermediate levels in *Eothenomys miletus* liver samples. Means with different superscript letters are significantly different (P < 0.05).

**Figure 9:**
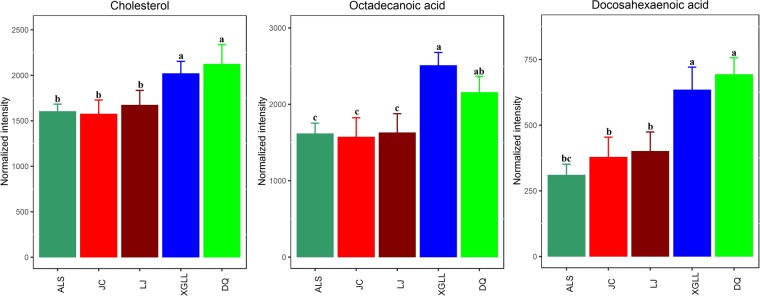
Lipid metabolite levels in *Eothenomys miletus* liver samples. Means with different superscript letters are significantly different (P < 0.05).

**Figure 10:**
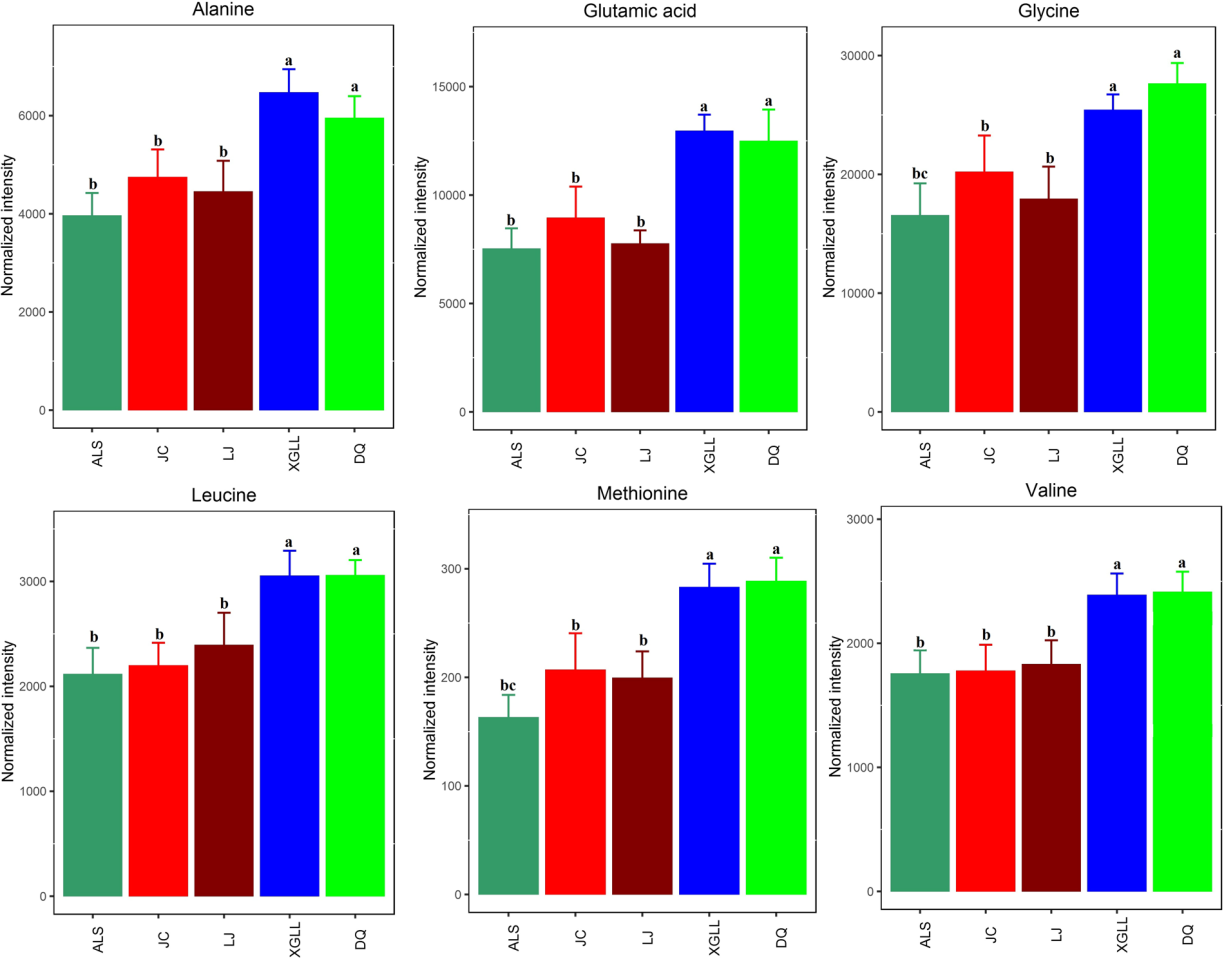
Amino acid metabolite levels in *Eothenomys miletus* liver samples. Means with different superscript letters are significantly different (P < 0.05).

**Figure 11:**
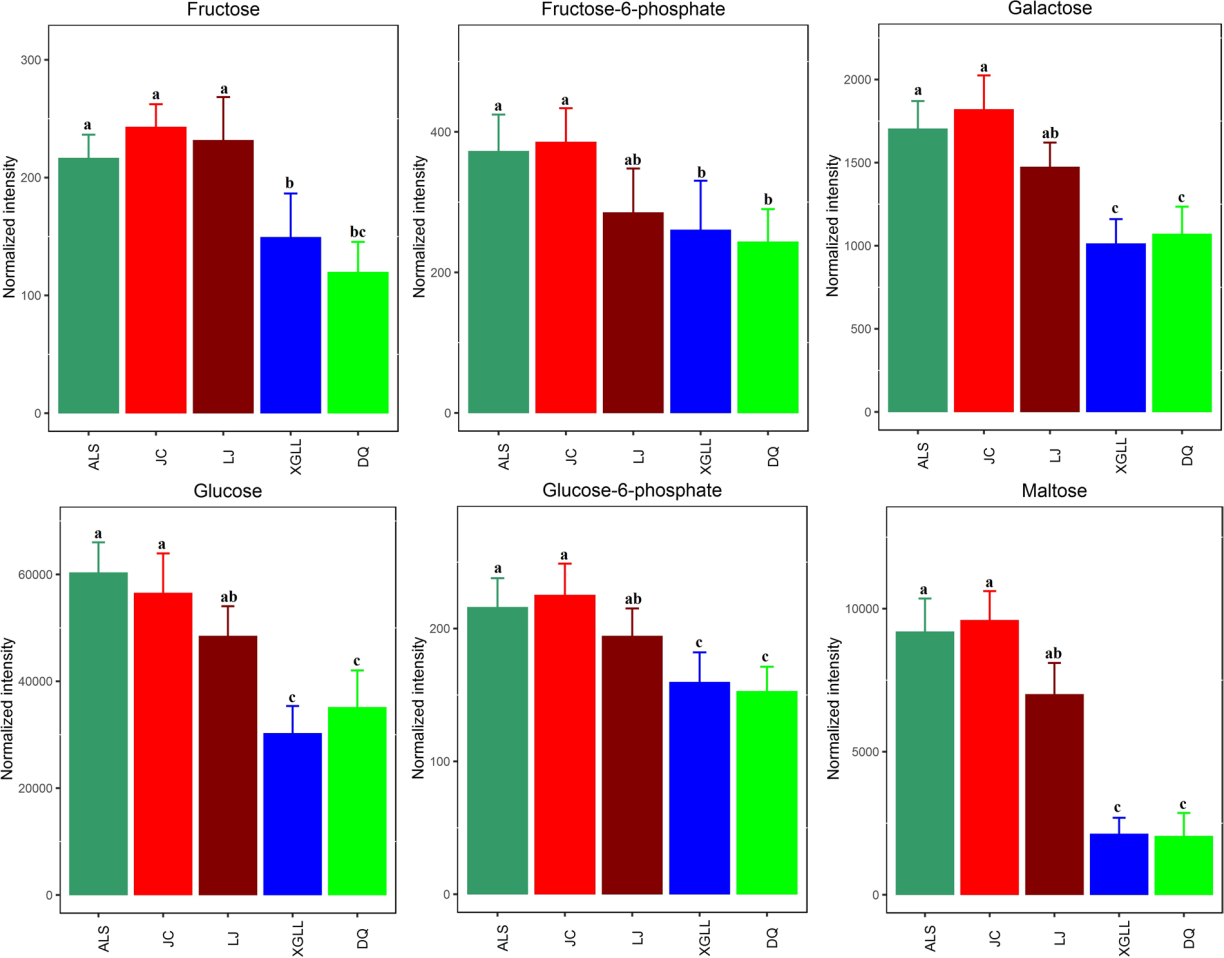
Glycolytic metabolite levels in *Eothenomys miletus* liver samples. Means with different superscript letters are significantly different (P < 0.05).

Heat maps of serum and liver metabolites and samples are shown in Fig. 5A,B. The concentrations of lipid metabolites (arachidonic acid, docosahexaenoic acid, hexadecanoic acid and nonadecanoic acid, Fig. 6) and amino acid metabolites (beta-alanine, lysine, phenylalanine and tyrosine, Fig. 7) in serum samples from DQ and XGLL were higher than those from LJ, JC and ALS. In addition, the concentrations of tricarboxylic acid (TCA) cycle intermediates (citric acid, fumaric acid and malic acid, Fig. 8), lipid metabolites (cholesterol, octadecanoic, and docosahexaenoic acid, Fig. 9) and amino acid metabolites (alanine, glutamic acid, glycine, leucine, methionine and valine, Fig. 10) in liver samples from DQ and XGLL were higher than those from the other three regions. However, the concentrations of glycolytic metabolites (fructose, fructose-6-phosphate, galactose, glucose, glucose-6- phosphate and maltose, Fig. 11) from DQ and XGLL were lower than those from other regions. Among the serum differential metabolites, fat metabolites and amino acid metabolites were negatively correlated with environmental temperature, but positively correlated with altitude. Among the liver differential metabolites, the intermediate products of TCA, fat metabolites and amino acid metabolites were positively correlated with environmental temperature, and negatively correlated with altitude, while the glycolytic metabolites were just the opposite.

**Figure 12:**
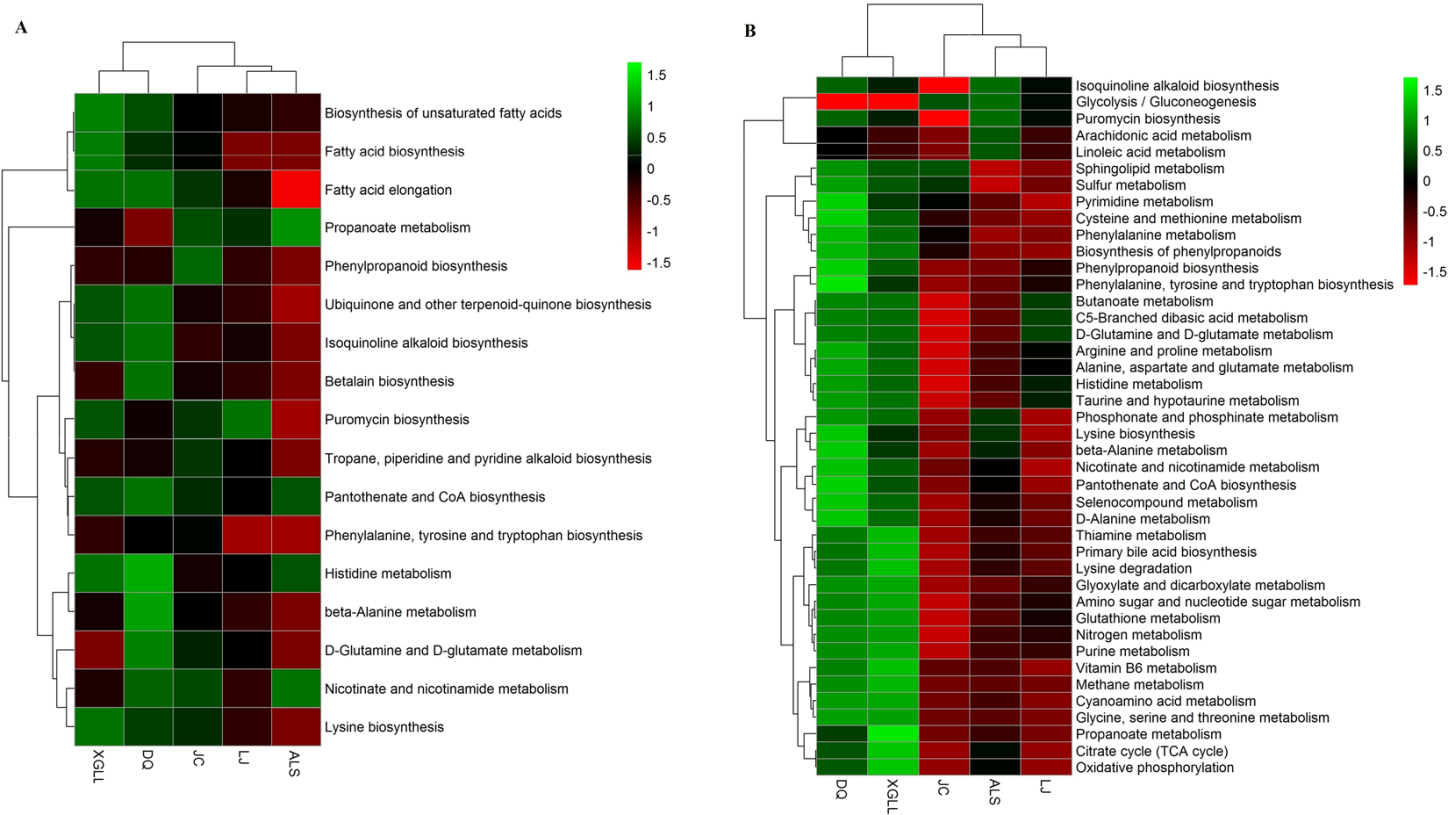
Heat map of serum (**A**) and liver (**B**) metabolic pathways and in *Eothenomys miletus*. Metabolite and population two-way clustering heat map. Colour depth represents metabolite contents in 5 populations of *Eothenomys miletus*. Green represents high content, red represents low content.

The serum metabolic pathway heat maps of *E*. *miletus* showed that metabolic pathway activities were distinct in different populations. The activities of the lipid and amino acid metabolic pathways were higher in the DQ and XGLL populations than in LJ, JC and ALS (Fig. 12A). The liver metabolic pathway heat maps also showed differences among the metabolic pathways in different populations. The activity of the TCA cycle and lipid and amino acid metabolic pathways were higher in the DQ and XGLL populations than in LJ, JC and ALS, while the activity of the glycolytic pathway was lower in DQ and XGLL than in other locations (Fig. 12B).

## Discussion

In small mammals, changes in body mass are influenced by environmental factors such as temperature and altitude^28,29^; which can cause variations in body mass during seasonal changes^30^. Previous studies have shown that *Microtus oeconomus* and *Lasiopodomys brandtii* decrease body mass and increase metabolic rate and food intake in winter^31^; seasonal changes in body mass have also been found in *Ochotona curzoniae*^32^. The liver is an important heat-producing organ in small mammals^28^, and liver mass in *Lasiopodomys brandtii* increases during cold acclimation^19^. In the present study, the DQ and XGLL regions had higher altitudes and lower annual average temperatures, while the LJ, JC and ALS regions had lower altitudes and higher annual average temperatures. Body mass in DQ and XGLL was lower than that in other three regions, indicating that temperature and altitude were key factors affecting body mass in *E*. *miletus*. With increasing altitude, the ambient temperature gradually decreased, which eventually led to lower body mass in *E*. *miletus*. However, liver wet mass was higher in DQ, probably because the ambient temperature was lower in that area; at lower temperatures, *E*. *miletus* requires additional thermogenesis to maintain thermoregulation, so the liver mass was greater.

Metabolomics can reflect plastic responses such as cold adaptation in animals^12^ and thus, this technique is often used to explore the adaptation mechanisms of organisms to environmental conditions such as temperature and altitude and determine the effects of high altitude and cold stress on animals^13,33,34^. Altitude and temperature differ across the Hengduan Mountains region, causing *E*. *miletus* to adapt to different environments. In the present study, the GC–MS method was used to examine serum and liver samples from five sites, and 88 and 86 metabolites were detected in the serum and liver, respectively. These metabolites included amino acids, organic acids, fatty acids, nucleotides, phosphoric acids, polyols, carbohydrates, amines and other substances. PLS-DA analysis of serum and liver metabolites found that samples from the higher-temperature, lower-altitude regions (ALS, JC and LJ) clustered together, while samples from the lower-temperature, higher-altitude regions (XGLL and DQ) were grouped together. The tree map of serum and liver metabolites showed similar results, suggesting that temperature and altitude may be the main environmental factors leading to the formation of two groups among the *E*. *miletus* from these five regions.

The TCA cycle is the key to energy in the organism, as it provides a link among sugar, lipid and protein metabolism^35^. TCA metabolites were increased in *Rattus norvegicus* in a long-term simulation of a high-altitude environment^33^. Lipid metabolism is an important way to produce thermogenesis^35^, and cold exposure increased fatty acid levels in *Rattus norvegicus*^36^. Higher altitude also had a significant effect on lipid metabolism in humans; phospholipid and free fatty acid levels increased when humans were exposed to higher altitudes^37^. Normal amino acid metabolism is an important basis for life activities. *Rattus norvegicus* exposed to a high-altitude environment showed an increase in amino acid metabolism^38^. Hypoxia leads to an increase in purine metabolites^39^ and higher-temperature environments reduced amino acid metabolites in *Gallus gallus domesticus*^40^. The main physiological function of carbohydrates is to provide the energy required for the organism^35^; temperature affects the glycolysis rate, and anaerobic glycolysis is the only mechanism to produce ATP in higher-altitude environments^41^. In the present study, the concentrations of lipid metabolites and amino acids in lower-temperature, higher-altitude regions (XGLL and DQ) were higher than those in higher-temperature, lower-altitude regions (ALS, JC and LJ). Furthermore, the metabolites of TCA, fatty acids, and amino acids in the liver in XGLL and DQ were higher than those in ALS, JC and LJ, while the concentrations of glycolytic metabolites in lower-temperature, higher-altitude areas were significantly lower than those in higher-temperature, lower-altitude areas. The DQ and XGLL locations have higher altitudes and lower annual average temperatures, so *E*. *miletus* living in these areas needs a large amount of sugar to produce energy to resist lower temperatures. Thus, the glycolytic metabolite contents were lower, while higher lipid metabolite and TCA cycle intermediate levels can help these animals increase their heat production capacity. In contrast, ALS, JC and LJ had higher temperatures, and the cold stress on the animals living there was less, so the concentrations of glycolytic metabolites were higher.

In conclusion, liver mass, the intermediate products of the TCA cycle, and the concentrations of lipid and amino acid metabolites were higher in *E*. *miletus* from DQ and XGLL. Body mass and glycolytic metabolites were greater in *E*. *miletus* from ALS, JC and LJ. All the above results showed that *E*. *miletus* in the Hengduan Mountains region adapted to changes in environmental temperature and altitude by adjusting body mass and serum and liver metabolite concentrations.
